# Transcriptomic response to heat stress among ecologically divergent populations of redband trout

**DOI:** 10.1186/s12864-015-1246-5

**Published:** 2015-02-21

**Authors:** Shawn R Narum, Nathan R Campbell

**Affiliations:** Columbia River Inter-Tribal Fish Commission, 3059-F National Fish Hatchery Road, Hagerman, ID 83332 USA

**Keywords:** Climate change, Oncorhynchus mykiss gairdneri, Redband trout, RNA-seq, Thermal adaptation, Transcriptome

## Abstract

**Background:**

As ectothermic organisms have evolved to differing aquatic climates, the molecular basis of thermal adaptation is a key area of research. In this study, we tested for differential transcriptional response of ecologically divergent populations of redband trout (*Oncorhynchus mykiss gairdneri*) that have evolved in desert and montane climates. Each pure strain and their F1 cross were reared in a common garden environment and exposed over four weeks to diel water temperatures that were similar to those experienced in desert climates within the species’ range. Gill tissues were collected from the three strains of fish (desert, montane, F1 crosses) at the peak of heat stress and tested for mRNA expression differences across the transcriptome with RNA-seq.

**Results:**

Strong differences in transcriptomic response to heat stress were observed across strains confirming that fish from desert environments have evolved diverse mechanisms to cope with stressful environments. As expected, a large number of total transcripts (12,814) were differentially expressed in the study (FDR ≤ 0.05) with 2310 transcripts in common for all three strains, but the desert strain had a larger number of unique differentially expressed transcripts (2875) than the montane (1982) or the F1 (2355) strain. Strongly differentiated genes (>4 fold change and FDR ≤ 0.05) were particularly abundant in the desert strain (824 unique contigs) relative to the other two strains (montane = 58; F1 = 192).

**Conclusions:**

This study demonstrated patterns of acclimation (i.e., phenotypic plasticity) within strains and evolutionary adaptation among strains in numerous genes throughout the transcriptome. Key stress response genes such as molecular chaperones (i.e., heat shock proteins) had adaptive patterns of gene expression among strains, but also a much higher number of metabolic and cellular process genes were differentially expressed in the desert strain demonstrating these biological pathways are critical for thermal adaptation to warm aquatic climates. The results of this study further elucidate the molecular basis for thermal adaptation in aquatic ecosystems and extend the potential for identifying genes that may be critical for adaptation to changing climates.

**Electronic supplementary material:**

The online version of this article (doi:10.1186/s12864-015-1246-5) contains supplementary material, which is available to authorized users.

## Background

Thermal adaptation is a widespread phenomenon in organisms that are exposed to variable and extreme environments. While some organisms may alter their distribution or behavior to avoid stressors and others may acclimate through physiological plasticity [1,2], many species evolve adaptive responses to local conditions over generations through natural selection [3-5]. Evolutionary adaptation to local environments has been demonstrated across a wide variety of taxa [6], and is expected to play a critical role for species with limited dispersal capabilities. However, few studies have identified the underlying molecular mechanisms that have led to conspecific adaptation to thermal conditions.

Molecular techniques such as RNA-seq [7] provide the opportunity to investigate transcriptional response to thermal stress and further identify mechanisms for thermal adaptation. Patterns of gene expression under heat stress are important to determining evolutionary adaptation among conspecific populations that occupy various environments. Multiple genes have been shown to be involved in heat tolerance across many species, including highly conserved heat shock proteins (hsps) that are upregulated under stressful conditions such as exposure to heat [8,9]. An adaptive heat shock response has additionally been shown to occur among conspecific populations that occupy variable environments [3,10]. However, many genes are known to have a role in regulating the effects of temperature and are likely to be involved in thermal adaptation [11,12]. Thus, RNA-seq provides the opportunity to investigate differential expression across the transcriptome and identify biological pathways involved in evolutionary response to thermal stress.

Redband trout (*Oncorhynchus mykiss gairdneri*) occupy highly variable environments including both montane and desert streams and have been shown to be locally adapted to these different environments [13]. Previous research has demonstrated an adaptive heat shock response among populations from different climates but also suggests that additional mechanisms are involved with thermal adaptation [14]. This species appears to have evolved a finely tuned heat shock response that likely requires additional genes to balance the short term (immediate cellular damage) and long term (fitness) costs associated with thermal stress. Given that oxygen delivery is limiting for fish under climate-related stressors [15], genes involved in oxygen transport are expected to play a significant role. Additionally, we expect that metabolic and immune pathways could be involved given the energy demands and potential for disease under chronic environmental stress [16,17]. Therefore, this study tests for molecular response to heat stress across the transcriptome of ecologically divergent populations of redband trout that have evolved under local climate regimes.

In this study, desert and montane strains of redband trout and their F1 crosses were tested for differential gene expression under heat stress in a common garden experiment. We tested for both acute and chronic stress response by quantifying gene expression in fish that experienced diel water temperatures similar to desert environments that peaked near their thermal maxima (~28.5 C) over the course of four weeks. Tissues were collected from each strain of redband trout at multiple time points (Day 1, 3, 7, and 28) to test for both acclimatization within each strain and evolutionary adaptation between strains. Results are quantified to confirm the role of hsp genes in these fish, but also identify additional genes and biological pathways that are regulated to balance the costs of stress response in populations that have evolved to desert environments.

## Results

### Sequence alignment to reference transcriptome

A total of 16 lanes for eight pooled libraries provided 2.30 billion quality filtered reads over all 72 samples. Read numbers ranged from 22.40-72.40 M per sample, with an overall mean of 31.96 M (Table 1). Read numbers were well balanced between treatment and control groups with 1.10 billion and 1.21 billion reads, respectively. Trimmed 60 bp reads were aligned with a minimum criterion of 95% identity to the reference transcriptome at an average of 24.1% and 7.53 M reads per sample. Mean percent alignment and mean number of aligned reads for each set of biological replicates ranged from 11.1-40.9% and 3.32-12.49 M, respectively (Additional file 1: Table S1). Reads aligned to a total of 25,128 unique contigs from the reference transcriptome. The mean percentage of aligned reads was not high (24.1%), but this experience is common for non-model species [18]. Trade-offs exist between aligning RNA-seq data to an existing reference transcriptome versus de-novo assembly [7,19,20]. We chose a conservative approach by aligning to a published reference transcriptome [21] that was assembled from multiple types of tissues under stress response which should be more representative than de-novo alignment with a single tissue type.

**Table 1:** **Summary data for redband trout samples including strain (LJ = Little Jacks Cr., K = Keithley Cr., LJxK = F1 crosses), temperature treatment (28°C treatment or 15°C control), sample day, sequencing reads (M = millions), and reference alignment statistics (transcriptome is abbreviated in column heading as Transc.)**

						**Mean**		**Mean**
				**Quality**	**Transcr.**	**Match (M)/**	**Transc.**	**Match (%)/**
Sample*	**Strain**	**Temp**	**Day**	**Reads (M)**	**Match (M)**	**Strain**	**Match (%)**	**Strain**
1	LJ	Trt-28C	1	28.1	4.6	7.0	16.2	23.1
2	LJ	Trt-28C	1	31.3	8.2		26.2	
3	LJ	Trt-28C	1	30.1	8.1		26.8	
4	LJ x K	Trt-28C	1	30.4	12.1	12.5	39.8	40.9
5	LJ x K	Trt-28C	1	27.6	11.6		42.1	
6	LJ x K	Trt-28C	1	33.6	13.7		40.9	
7	K	Trt-28C	1	32.4	13.1	12.1	40.4	40.3
8	K	Trt-28C	1	31.4	13.4		42.5	
9	K	Trt-28C	1	25.9	9.9		38.0	
10	LJ	Trt-28C	3	40.6	14.1	10.8	34.7	30.2
11	LJ	Trt-28C	3	34.8	9.8		28.3	
12	LJ	Trt-28C	3	30.5	8.4		27.7	
13	LJ x K	Trt-28C	3	31.3	9.1	9.0	29.0	26.1
14	LJ x K	Trt-28C	3	30.2	6.9		23.0	
15	LJ x K	Trt-28C	3	41.7	10.9		26.3	
16	K	Trt-28C	3	27.3	9.6	7.0	35.2	24.4
17	K	Trt-28C	3	28.3	7.6		27.0	
18	K	Trt-28C	3	34.3	3.8		11.0	
19	LJ	Trt-28C	7	29.3	12.4	9.1	42.4	29.2
20	LJ	Trt-28C	7	31.5	9.4		29.9	
21	LJ	Trt-28C	7	35.1	5.4		15.5	
22	LJ x K	Trt-28C	7	28.5	10.8	10.5	37.9	38.2
23	LJ x K	Trt-28C	7	26.4	9.9		37.6	
24	LJ x K	Trt-28C	7	28.0	10.9		39.1	
25	K	Trt-28C	7	37.4	6.8	5.2	18.2	16.6
26	K	Trt-28C	7	28.7	5.0		17.3	
27	K	Trt-28C	7	27.8	4.0		14.2	
28	LJ	Trt-28C	28	22.8	6.4	5.6	28.2	20.6
29	LJ	Trt-28C	28	31.2	7.5		24.0	
30	LJ	Trt-28C	28	29.6	2.8		9.6	
31	LJ x K	Trt-28C	28	43.0	0.5	3.7	1.1	14.9
32	LJ x K	Trt-28C	28	24.1	4.1		16.9	
33	LJ x K	Trt-28C	28	24.3	6.4		26.5	
34	K	Trt-28C	28	26.4	6.3	6.0	23.7	23.0
35	K	Trt-28C	28	25.4	7.3		28.7	
36	K	Trt-28C	28	26.8	4.5		16.6	
37	LJ	Con-15C	1	23.8	7.4	6.9	31.2	27.0
38	LJ	Con-15C	1	24.7	6.7		27.3	
39	LJ	Con-15C	1	29.4	6.6		22.5	
40	LJ x K	Con-15C	1	25.0	6.5	10.2	25.9	25.0
41	LJ x K	Con-15C	1	65.0	15.3		23.5	
42	LJ x K	Con-15C	1	34.2	8.7		25.6	
43	K	Con-15C	1	22.4	5.3	6.9	23.7	26.0
44	K	Con-15C	1	27.1	8.3		30.6	
45	K	Con-15C	1	29.6	7.0		23.7	
46	LJ	Con-15C	3	24.2	8.1	5.4	33.5	19.7
47	LJ	Con-15C	3	34.4	3.7		10.9	
48	LJ	Con-15C	3	30.0	4.4		14.8	
49	LJ x K	Con-15C	3	46.3	0.7	3.3	1.6	11.1
50	LJ x K	Con-15C	3	27.0	3.6		13.4	
51	LJ x K	Con-15C	3	30.6	5.6		18.4	
52	K	Con-15C	3	26.7	8.3	5.8	31.0	21.2
53	K	Con-15C	3	28.0	4.3		15.4	
54	K	Con-15C	3	28.5	4.9		17.1	
55	LJ	Con-15C	7	24.3	7.1	6.0	29.4	24.0
56	LJ	Con-15C	7	26.2	7.0		26.9	
57	LJ	Con-15C	7	25.0	4.0		15.9	
58	LJ x K	Con-15C	7	33.2	6.7	4.3	20.2	13.8
59	LJ x K	Con-15C	7	32.6	2.9		8.9	
60	LJ x K	Con-15C	7	27.3	3.4		12.4	
61	K	Con-15C	7	25.6	5.0	10.8	19.4	23.4
62	K	Con-15C	7	27.8	5.9		21.0	
63	K	Con-15C	7	72.7	21.7		29.9	
64	LJ	Con-15C	28	35.0	12.5	9.5	35.6	25.9
65	LJ	Con-15C	28	41.1	7.1		17.4	
66	LJ	Con-15C	28	36.1	8.9		24.6	
67	LJ x K	Con-15C	28	31.5	5.9	8.3	18.6	21.2
68	LJ x K	Con-15C	28	54.7	7.1		12.9	
69	LJ x K	Con-15C	28	37.3	11.9		32.1	
70	K	Con-15C	28	44.4	0.3	4.9	0.7	13.4
71	K	Con-15C	28	41.5	6.9		16.5	
72	K	Con-15C	28	32.3	7.4		22.9	
mean	--	--	--	31.96	7.53	7.5	24.1	24.1

*Each sample includes 3 pooled RNA samples from the same rearing tank.

**Figure 1:**
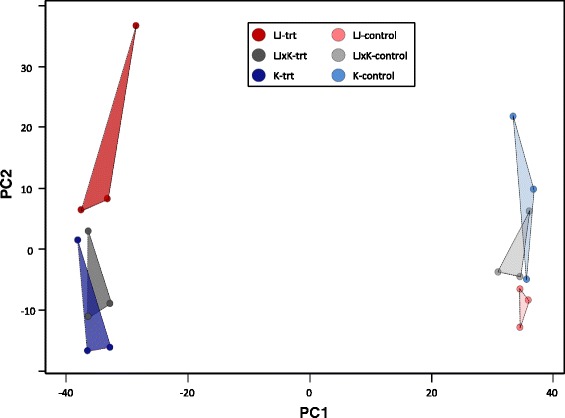
**Principal components analysis of overall transcriptome expression.** Results for 18 samples of redband trout collected either at first exposure to heat stress up to 28°C (darker shades) or on the same day from 15°C control temperature (lighter shades). Samples are color coded by their environment: desert strain (red; LJ = Little Jacks Cr.), F1 crosses (gray; LJxK), montane strain (blue; K = Keithley Cr.).

Principal component analyses of overall gene expression data clearly showed that samples clustered by treatment or control condition and also that distinct clusters were present for the desert and montane strains, but the cluster for the F1 strain overlapped with the montane strain (Figure 1).

### Differential expression

**Figure 2:**
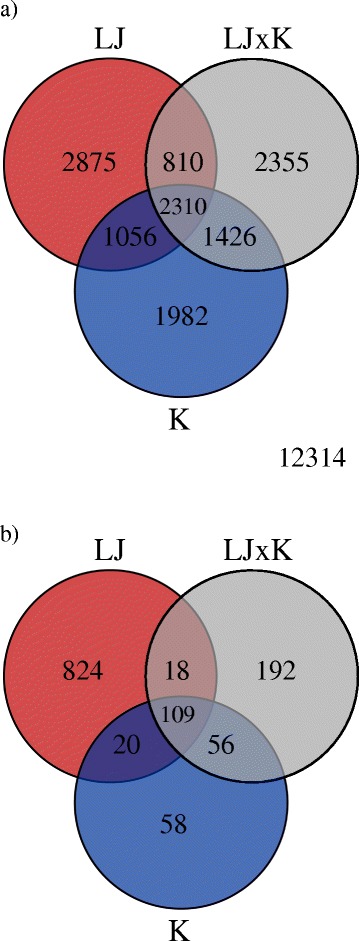
**Venn diagrams of differentially expressed genes.** Results of each strain of redband trout for **a)** all significant transcripts (FDR ≤ 0.05); and **b)** strongly differentiated transcripts (>4 fold change and FDR ≤ 0.05). Circles are color coded to represent fish by their environment: desert strain (red; LJ = Little Jacks Cr.), F1 crosses (gray; LJxK), montane strain (blue; K = Keithley Cr.). For a) there were 12,314 genes that were not statistically significant at either level and are listed outside of the circles on the Venn diagram.

**Figure 3:**
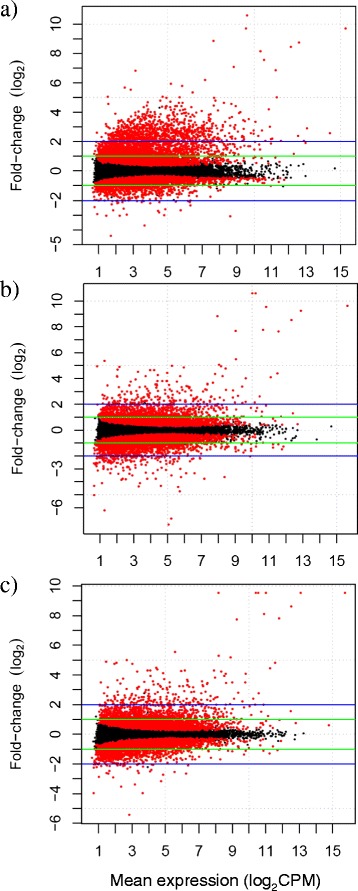
**Adaptation: differential expression for each strain of redband trout across all time periods of heat stress versus fish held at control temperatures.** Results for **a)** desert strain from Little Jacks Cr. **b)** F1 crosses; and **c)** montane strain from Keithley Cr. Genes that are significantly differentiated (FDR ≤ 0.05) are in red and those that are not significant are in black. On a log_2_ scale, the green lines show genes that are ≥ 2 fold, and the blue lines designate genes that are ≥ 4 fold. The x-axis is the mean expression of each gene in counts per million reads (CPM) on a log_2_ scale.

Differences in gene expression were highly significant within each strain (Figure 2a), with 7,051 significant genes for the desert strain (4,238 upregulated, 2,813 downregulated), 6,906 for F1 crosses (3,375 upregulated, 3,531 downregulated), and 6,774 for montane (3,499 upregulated, 3,275 downregulated). As expected, a large number of total transcripts (12,814) were differentially expressed in the study (FDR ≤ 0.05) with 2,310 transcripts occurring in common among all three strains, but the desert strain had a larger number of unique differentially expressed transcripts (2,875) than the montane (1,982) or the F1 (2,355) strain (Figure 2a). Strongly differentiated genes (>4 fold change and FDR ≤ 0.05; Figure 2b) were highly abundant in the desert strain (824 unique transcripts; Additional file 1: Table S1) relative to the other two strains (montane = 58; Additional file 2: Table S2; F1 = 192; Additional file 3: Table S3), particularly in upregulated transcripts (Figure 3a-c).

**Figure 4:**
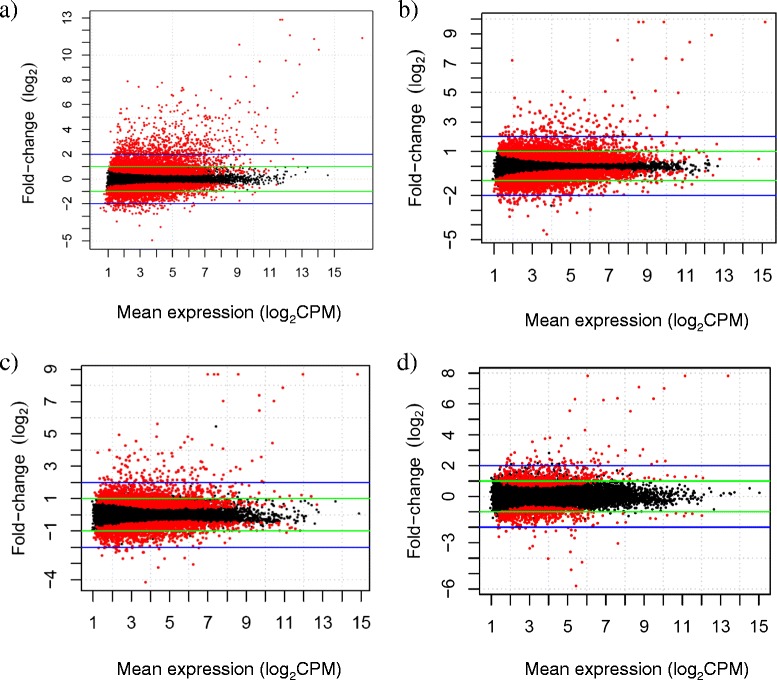
**Acclimation: differential expression at each time period of heat stress versus fish held at control temperatures across all strains.** Results for **a)** Day 1 of heat stress treatment; **b)** Day 3 of heat stress treatment; **c)** Day 7 of heat stress treatment; and **d)** Day 28 of heat stress treatment. The number of significant genes was 7,833 at Day 1 (4,058 upregulated, 3,775 downregulated), 6,408 at Day 3 (3,344 upregulated, 3,064 downregulated; 18.2% decrease from Day 1), 3,624 at Day 7 (1,958 upregulated, 1,666 downregulated; 53.7% decrease from Day 1), and 1,269 at Day 28 (719 upregulated, 550 downregulated; 83.8% decrease from Day 1). Genes that are significantly differentiated (FDR ≤ 0.05) are in red and those that are not significant are in black. On a log_2_ scale, the green lines show genes that are ≥ 2 fold, and the blue lines designate genes that are ≥ 4 fold. The x-axis is the mean expression of each gene in counts per million reads (CPM) on a log_2_ scale.

Differences in gene expression were also observed over time as the number of significant genes consistently decreased with more days of exposure to temperature stress (Figure 4). The number of significant genes was 7,833 at Day 1 (4,058 upregulated, 3,775 downregulated), 6,408 at Day 3 (3,344 upregulated, 3,064 downregulated; 18.2% decrease from Day 1), 3,624 at Day 7 (1,958 upregulated, 1,666 downregulated; 53.7% decrease from Day 1), and 1,269 at Day 28 (719 upregulated, 550 downregulated; 83.8% decrease from Day 1). This trend was consistent with the expectation that the stress response would become reduced with chronic exposure to heat stress.

**Figure 5:**
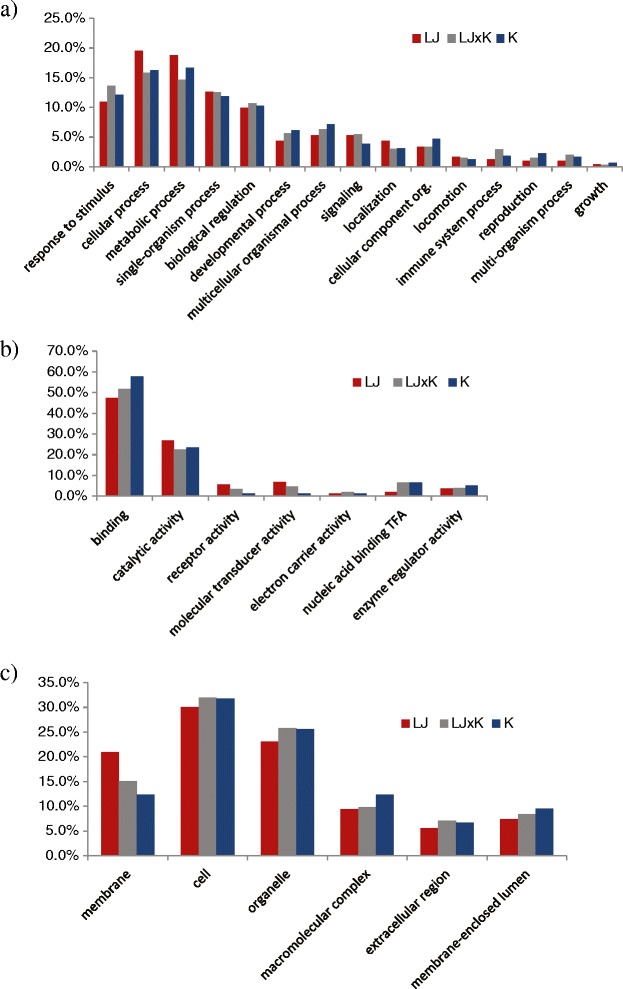
**Gene ontology (GO) annotation for transcripts that were strongly differentiated expressed in each strain (>4 fold change and FDR ≤ 0.05).** Results shown for level 2 categories for **a)** Biological process (“org.” = organization); **b)** Molecular function (“TFA” = transcription factor activity); **c)** Cellular component. Bars are color coded to represent fish by their environment: desert strain (red; LJ = Little Jacks Cr.), F1 crosses (gray; LJxK), montane strain (blue; K = Keithley Cr.).

Gene ontology and enrichment with Blast2GO revealed that strongly differentiated genes in each strain (>4 fold change and FDR ≤ 0.05) included several categories for each of biological processes, molecular function, and cellular components (Figure 5a-c; Additional files 4, 5 and 6: Tables S4-6). Within biological process, there were a total of 18 pathway categories at level 2 gene ontology, but nearly 70% of the genes were included in five categories: cellular process (mean = 17.2%), metabolic process (mean = 16.7%), response to stimulus (mean = 12.2%), single-organism process (mean = 12.4%), and biological regulation (mean = 10.3%; Figure 5a). Within molecular function, there were a total of 11 pathway categories at level 2 gene ontology with over 75% of the genes in two categories: binding (mean = 52.3%), and catalytic activity (mean = 24.3%; Figure 5b). Within cellular components, there were a total of 10 pathway categories at level 2 gene ontology, with over 80% of the genes included in four categories: cell (mean = 31.3%), organelle (mean = 24.8%), membrane (mean = 16.1%), and macromolecular complex (mean = 10.5%; Figure 5c). Finally, many of the genes that were found to be differentially expressed relative to control fish in all strains and time points were stress response genes such as various hsp transcripts.

Patterns of gene expression for each strain over time were compared with results from qPCR assays for heat shock genes and were highly consistent with either RNA-seq or qPCR data (Additional file 7: Figure S1). Specifically, expression patterns showed that heat shock genes were significantly lower for the desert strain at Day 1 for all hsp genes and all strains had decreased gene expression from Day 3 through the remainder of the experiment as shown previously [14].

## Discussion

Since thermal stress has broad biological effects on organisms, transcriptional response is expected to be highly diverse across several genes in ectothermic species such as redband trout. This study confirms that numerous genes are differentially expressed in redband trout under heat stress, and several pathways are involved. However, there were key pathways that contained a large proportion of differentially expressed transcripts including response to stimulus, metabolic processes, cellular processes, molecular binding function, and cell membrane function. These pathways correspond well with previous studies that demonstrate these as critical physiological components involved with of aquatic ectotherms exposed to elevated water temperatures [17,22]. In particular, several physiological studies have linked thermal tolerance with aerobic scope and emphasize the role of metabolic processes in thermal adaptation (e.g., [22-24]). The larger number of differentially expressed genes in the desert strain versus the other two strains suggests that a complex combination of genes has evolved for redband trout in their desert environment. It is also possible that a few genes with large pleiotropic effects could be responsible for the pattern observed.

Evidence for acclimation to heat stress was extensive as the number of differentially expressed transcripts decreased by 83.7% from Day 1 to Day 28. Results from this study elaborate on previous findings in redband trout that stress response genes are highly upregulated when exposed to heat stress [25,14]. Multiple heat shock genes (e.g., hsp70, hsp90, hsp47) were differentially expressed in all strains and time periods. However, an acclimation effect was evident as expression levels decreased over time in all strains. This is consistent with theories of acclimation to heat stress where organisms are able to moderate their heat shock response over time, as opposed to initial exposure where immediate survival is a priority [26]. However, the current results demonstrate that acclimation within strains occurs throughout much of the transcriptome and is not limited to heat shock genes.

More importantly, this study demonstrates that adaptive patterns of expression have evolved in ecologically divergent populations of this species. Results from Narum et al. [14] specifically highlight the adaptive response of heat shock genes in redband trout, with lower hsp gene expression observed in desert versus montane strains. Results in heat shock genes from the current RNA-seq data corroborate the previous qPCR results and emphasize that warm adapted natural populations are likely to have evolved a specialized heat shock response that reduces physiological costs of hsp production. This result is consistent with the adaptive heat shock response observed in natural populations of other organisms such as killifish (*Fundulus heteroclitus*; [27]) and *Drosophila buzzatii* [10]. This remains an important finding of this study and provides clarification regarding evolutionary adaptation of hsp gene expression in heat tolerant populations. However, many recent studies indicate that complex mechanisms are involved in thermal adaptation of aquatic ectotherms beyond heat shock response (e.g., [22,28,29]. Indeed, this study of the transcriptome revealed adaptive patterns in metabolic and cellular process genes that suggest desert fish are more efficient at supporting these pathways than montane fish under heat stress, and chronic exposure may cause failure of these genes to be expressed in montane and F1 crosses and suggests that some critical physiological functions become limited in these strains over time. Previous studies suggest that metabolic pathways may be particularly important since metabolic energy stores are positively correlated with physiological function and swimming behavior in thermally adapted redband trout [16,17]. The general pattern observed across the transcriptome for F1 crosses indicates intermediate gene expression consistent with additive variation, but there may be a maternal or dominant effect at some genes since more differentially expressed genes were shared between the F1s and the maternal montane strain than the desert strain.

A variety of immediate and long-term anthropogenic disturbances such as habitat destruction and climate change have negative impacts on freshwater fish [30,31] and the need to understand mechanisms for thermal adaptation in these organisms is critical. Many fishes have already been extirpated from large portions of their historical range (e.g., [32]) and the effects of climate change are expected to further alter species’ range, phenology, and persistence [32-35]. Genomic and physiological mechanisms for thermal adaptation can be important tools for conservation measures to enable long-term viability of wild populations [36-38]. Specifically, this study helps to further identify genomic tools such as genetic screening with candidate genes that may be integrated with measurements of cardiac function [22] in order to screen broadly across species’ range to predict the potential for adaptation under scenarios of climate change [39].

## Conclusions

This study demonstrates that redband trout from a desert climate have a much larger number of strongly differentially expressed genes than montane and F1 strains in response to heat stress, suggesting that a combination of genes has evolved for redband trout to adapt in their desert environment. Recent studies of physiological adaptation in aquatic ectotherms indicate that intraspecific thermal tolerance is set by limitations in aerobic performance, specifically the upper limit of heart rate to deliver oxygen to tissues (e.g., [15,22,23]). This is due to temperature dependent oxygen limitation in aquatic environments, a theory that has been well supported in many organisms [40]. In order to support this increase in cardiac performance, redband trout would need to differentially regulate genes from multiple pathways including those observed in this study (e.g., metabolic pathways). However, further studies that specifically link individual gene expression [41] with physiological functions such as aerobic scope and heart rate are needed to further elucidate the specific mechanisms involved with thermal adaptation in this species. Development of a reference transcriptome and genome that are more specific to this subspecies of *O. mykiss* would also provide better annotation of genomic architecture of various traits such as thermal adaptation.

## Methods

### Redband trout populations and thermal stress experiments

To investigate thermal acclimation and adaptation of redband trout (*O. mykiss gairdneri*) from desert and montane populations, fry of approximately four months of age from each environment and their F1 crosses were exposed to diel temperature cycles (peaking at 28°C) over a 4-week period in a controlled setting. Gill tissues were collected from euthanized individuals on day 1, 3, 7 and 28 to quantify mRNA expression across the transcriptome. Gill tissue was used for this study since oxygen transport and osmoregulation are expected to play important functional roles in thermal adaptation.

Gametes and fry were collected from two ecologically divergent populations: one from a desert climate stream Little Jacks Cr. (LJ), and one from a montane climate stream Keithley Cr. (K), both located in Idaho, USA. In order to create F1 crosses, gametes from each strain were cross fertilized and reared in laboratory incubators. F1s were included to investigate additive genetic variation associated with response to heat stress. The two sites were chosen for study based on previous tests of redband trout from six desert and six montane streams that demonstrated that Little Jacks Cr. fish were adapted to a desert climate and Keithley Cr. was a typical montane stream population [13]. Gametes were fertilized to produce half-sibling progeny representing three distinct strains: one of pure desert strain (LJ), one of pure montane strain (K), and the F1 crosses (LJ males × K females). Fry were reared in constant 15°C spring water until they reached an average weight of 2 g, then each strain was divided into treatment and control groups. Three replicate tanks were used for all treatment and control groups for each strain (3 strains × 2 treatments × 3 replicates equals a total of 18 tanks) with an average of 45 fish per tank. Fish were fed a diet of Soft Moist pellets (Rangen Inc.) to satiation twice per day, and photoperiod was fixed at 14 h light and 10 h darkness. Fish in recirculating treatment tanks experienced diel temperature cycles over 6 weeks that reached a maximum of 28.5°C in the afternoon and a minimum of 17.0°C at night (mean temperature gradient of ~1.5°C per hour; Additional file 7: Figure S1, Supporting information), while fish in control tanks were held at a constant temperature of 15°C spring water. All experimental protocols were approved in advance by the University of Idaho’s Institutional Animal Care and Use Committee (Protocol #201025).

### RNA-seq library prep and Illumina sequencing

Total RNA was isolated from approximately 5 mg of gill tissue from individual fish using Qiagen RNeasy kits. RNA was normalized to 100 ng/μL and equal volumes of RNA from three fish from each tank were pooled for a total of 72 libraries (18 tanks × 4 time periods each; Table 1). The Ribo-Zero™ Magnetic Gold Kit (Epicentre) was used to deplete the samples of ribosomal RNA (rRNA) which constitutes a large proportion of the total RNA. Ribosomal RNA depleted samples were purified using the ethanol precipitation method suggested in the Ribo-Zero Kit protocol and resuspended in 20 μL of RNAse free water. RNA-seq libraries were prepared using 4.75 μL of template material using the strand-specific library preparation kit ScriptSeq™ v2 RNA-Seq Kit (Epicentre, Madison WI USA). The tagged cDNA constructs were purified using Qiagen MinElute columns and sample specific index sequences were added during PCR [95°C – 1 m; (95°C – 30s; 55°C – 30s; 68°C – 3 m) × 15; 68°C – 7 m; 4°C – hold] using ScriptSeq (Epicentre) index PCR primers. Each amplified library was then purified using Agencourt AMPure XP magnetic beads and eluted with 20uL of nuclease free water. Dilutions (1:2000) of the purified and indexed libraries were then quantified by qPCR using a ABI-7900 instrument (Life Technologies), Power-Sybr master mix (Life Technologies), standard Illumina (P5, P7) primers and an Illumina PhiX library as a standard. The indexed libraries were normalized to 10 nM concentration in Tris-EDTA (pH 8.0) buffer with 0.1% Tween-20 and combined for sequencing (8 pooled libraries with nine samples, each with ScriptSeq index sequences 1–9). Each of the pooled libraries was sequenced in two lanes of a single read 100 bp flow cell on an Illumina HiSeq 1500 instrument for a total of 16 lanes. Each lane of data was demultiplexed by index sequence and reads were combined from both lanes for each sample. The average number of reads per sample after quality filtering was 31.96 M and ranged from 22.40 – 72.70 M. Raw data was submitted to NCBI’s short read archive (SRA; entry GSE53907).

### Sequence alignment to reference transcriptome

Raw sequencing data was aligned to a reference transcriptome for rainbow trout designed for stress response [21] using the program Bowtie [42]. Parameters for Bowtie were set to exclude the first 10 bases and last 30 bases of sequencing data leaving 60 bases of high quality sequence for alignment. Both the forward (+) and reverse complement (−) of each reference transcript were considered for alignment since the reference transcriptome was assembled from non-directional sequence data. The best single match to the reference transcriptome was returned provided it had no more than 3 mismatches across the 60 bases (95% identical).

Output data from Bowtie was condensed by counting matches to each of the reference transcript contigs for both + and - orientation. Since the library preparation process generates directional constructs (strand-specific library preparation), we expected legitimate alignments to match only one of the two orientations of each reference transcript. Indeed, we found that our library reads predominantly matched only 1 of the 2 strand orientations (>90% of reads). Therefore, read alignments to the minor strand were considered mis-assigned and excluded from the data set. Finally, since the reference transcriptome included several sequence variants (contigs) for many putative genes, combined read counts from contigs within each gene were utilized for differential expression analyses.

### Differential expression analyses

Tests for differential expression were completed using the edgeR Bioconductor package [43]. Differential expression analyses were done to test for two processes involved in heat stress response: 1) acclimatization to chronic heat stress over time, and 2) evolutionary adaptation of the specific strains. To test for acclimatization, differential expression was tested separately for each time period (Day 1, 3, 7, 28) with all strains in the model. To test for evolutionary adaptation, differential expression was tested separately for each strain (LJ, K, LJxK) with all time periods in the model. Each model included the additional factor of condition (treatment or control) and three biological replicates. Genes that were not expressed in either condition were removed; specifically only genes with at least two counts per million reads in at least nine samples were kept for further analyses. Gene counts were normalized with the trimmed mean of ‘M’ values (TMM) method in edgeR as this has been shown to be one of the most reliable methods for this purpose in RNA-seq studies [44]. Sample metadata and normalized gene expression data was submitted to NCBI’s Gene Expression Omnibus (GEO; entry GSE53907). As suggested by McCarthy et al. [45], genewise dispersion and a general linear model (GLM) were used for tests of differential expression. Genewise dispersion estimates deprioritize genes with inconsistent results and allow the main analysis to focus on changes that are consistent between biological replicates. The GLM accounts for the multifactor design of this study. A false discovery rate (FDR at 0.05; [46]) was applied to account for multiple tests of differentially expressed genes. In order to validate patterns of gene expression in the RNA-seq data, results were compared to quantitative PCR (qPCR) data for multiple heat shock genes with Sybr Green on an ABI 3730 instrument as detailed in Narum et al. [14]. Significantly regulated contigs in each strain were annotated in Blast2GO with a blastx minimum e-value set to 1.0E-06 [47].

### Availability of supporting data

Raw data was submitted to NCBI’s short read archive (SRA; entry GSE53907). Sample metadata and normalized gene expression data was submitted to NCBI’s Gene Expression Omnibus (GEO; entry GSE53907).

## Additional files

Additional file 1: Table S1. Complete list of genes for Little Jacks Cr. redband trout and subsets of significant genes at differing levels of statistical significance (FDR ≤ 0.05; > 4 fold change and FDR ≤ 0.05). Gene identification number is provided from the reference transcriptome [18], FC = fold change, CPM = counts per million, LR = likelihood ratio, FDR = false discovery rate. Additional file 2: Table S2. Complete list of genes for F1 crosses of redband trout (Little Jacks Cr. x Keithley Cr.) and subsets of significant genes at differing levels of statistical significance (FDR ≤ 0.05; > 4 fold change and FDR ≤ 0.05). Gene identification number is provided from the reference transcriptome [18], FC = fold change, CPM = counts per million, LR = likelihood ratio, FDR = false discovery rate. Additional file 3: Table S3. Complete list of genes for Keithley Cr. redband trout and subsets of significant genes at differing levels of statistical significance (FDR ≤ 0.05; > 4 fold change and FDR ≤ 0.05). Gene identification number is provided from the reference transcriptome [18], FC = fold change, CPM = counts per million, LR = likelihood ratio, FDR = false discovery rate. Additional file 4: Table S4. Annotated genes for Little Jacks Cr. redband trout that were strongly differentially expressed (>4 fold change and FDR ≤ 0.05). SeqName is the gene identification number from the reference transcriptome [18], and GO-Groups are abbreviated as P – Process, F – Function, C – Cellular. Additional file 5: Table S5. Annotated genes for F1 crosses of redband trout (Little Jacks Cr. x Keithley Cr.) that were strongly differentially expressed (>4 fold change and FDR ≤ 0.05). SeqName is the gene identification number from the reference transcriptome [18], and GO-Groups are abbreviated as P – Process, F – Function, C – Cellular. Additional file 6: Table S6. Annotated genes for Keithley Cr. redband trout that were strongly differentially expressed (>4 fold change and FDR ≤ 0.05). SeqName is the gene identification number from the reference transcriptome [18], and GO-Groups are abbreviated as P – Process, F – Function, C – Cellular. Additional file 7: Figure S1. Gene expression patterns of heat shock genes as estimated by RNA-seq and qPCR methods for a) hsp70, b) hsp47, and c) hsp 90.
