# Supplementary material for: Transcriptomic response to heat stress among ecologically divergent populations of redband trout
Source: BMC Genomics. 2015 Feb 21;16(1):103. doi: 10.1186/s12864-015-1246-5 (PMC4337095; doi:10.1186/s12864-015-1246-5)

Figure S1. Gene expression patterns of heat shock genes as estimated by RNA-seq and qPCR methods for a) hsp70, b) hsp47, and c) hsp 90.

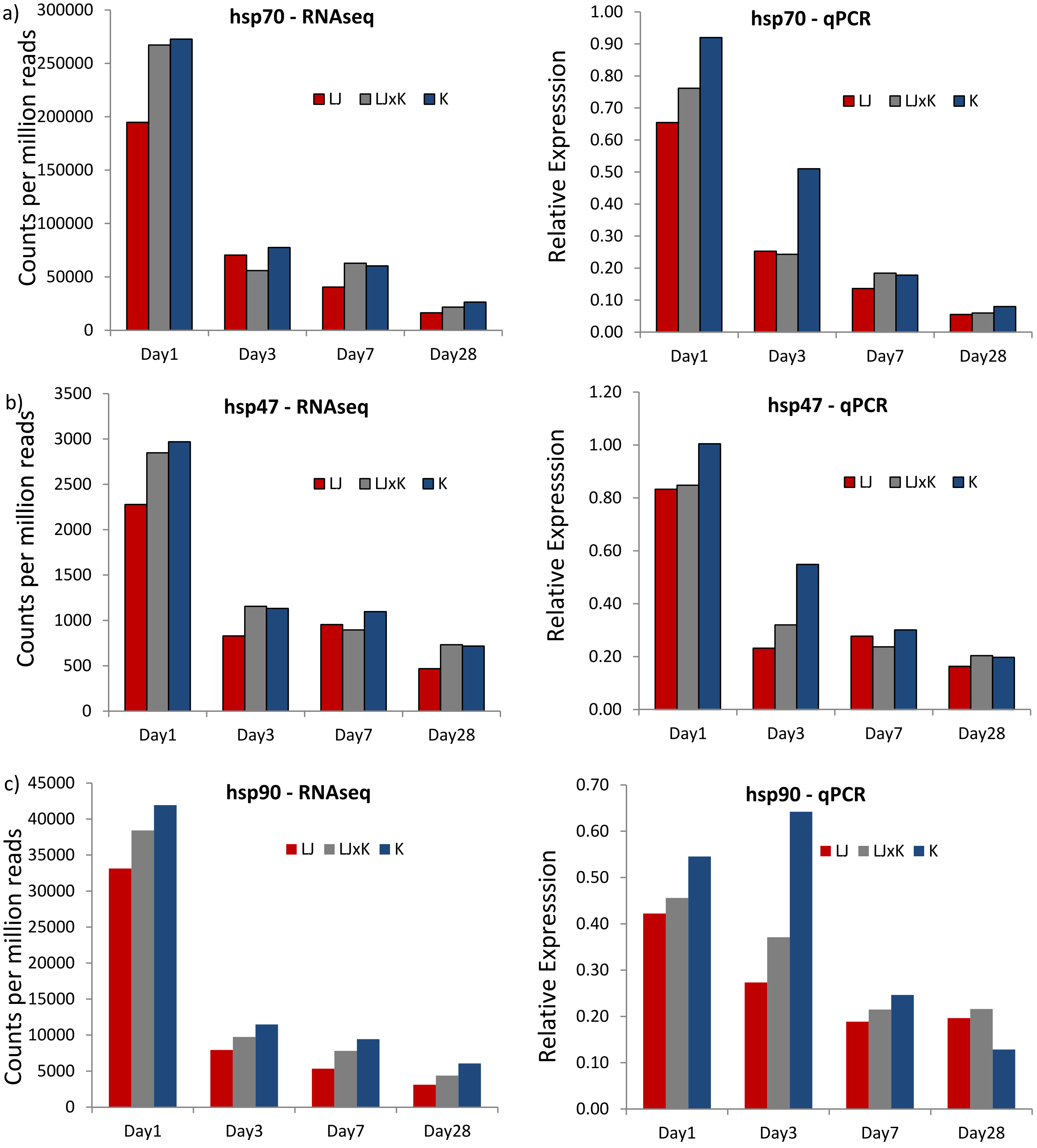

Supplement: Additional file 7: Figure S1. — Gene expression patterns of heat shock genes as estimated by RNA-seq and qPCR methods for a) hsp70, b) hsp47, and c) hsp 90. [file 12864_2015_1246_MOESM7_ESM.pdf]
